# Role of steroid hormones and morphine treatment in the modulation of opioid receptor gene expression in brain structures in the female rat

**DOI:** 10.1186/s40064-015-1021-8

**Published:** 2015-07-16

**Authors:** Wesley Soares Cruz, Lucas Assis Pereira, Luana Carvalho Cezar, Rosana Camarini, Luciano Freitas Felicio, Maria Martha Bernardi, Elizabeth Teodorov

**Affiliations:** Instituto de Ciências da Saúde, Universidade Paulista, UNIP, Dr. Bacelar, São Paulo, CEP 04026-002 Brazil; Instituto de Ciências Biomédicas, Universidade de São Paulo, Av. Prof. Lineu Prestes 2415, Cidade Universitária, SP CEP 05508-900 Brazil; Faculdade de Medicina Veterinária e Zootecnia, Universidade de São Paulo, Av. Prof. Dr. Orlando Marques de Paiva, 87, Cidade Universitária, CEP 05508 270 Brazil; Centro de Matemática, Computação e Cognição, Universidade Federal do ABC, Av. Dos Estados, 5001, Santo André, CEP 09210-971 Brazil; Av dos Estados, 5001, Santo André, SP CEP 09210-970 Brazil

**Keywords:** Opioid, Estrogen, Progesterone, Striatum, Hypothalamus, Periaqueductal gray

## Abstract

This study determined the effects of acute treatment with morphine on the expression of the *Oprm1*, *Oprk1*, and *Oprd1* genes (which encode μ, κ, and δ receptors, respectively) in the striatum, hypothalamus, and periaqueductal gray (PAG) in ovariectomized female rats treated with estrogen. Ovariectomized female rats were divided into five equal groups. Two groups received estrogen (50 µg/kg, 54 h before testing) and saline (ES group) or 3.5 mg/kg morphine (EM group) 2 h before euthanasia. The SS group received saline solution 54 and 2 h before the experiments. The SM group received saline 54 h and 3.5 mg/kg morphine 2 h before the experiments. The W group remained undisturbed. The genes expression were evaluated. *Oprm1* and *Oprk1* expression were activated, respectively, in the hypothalamus and PAG and in the striatum and PAG by morphine only in estrogen-treated animals. *Oprd1* expression in the hypothalamus and PAG was activated by morphine in both estrogen-treated and -nontreated animals. The *Oprm1* and *Oprk1* gene response to morphine might depend on estrogen, whereas the *Oprd1* gene response to morphine might not depend on estrogen, supporting the hypothesis of a functional role for ovarian hormones in opioid receptor-mediated functional adaptations in the female brain.

## Background

Female gonadal hormones have an important impact on many brain functions and behaviors related to reproduction and neurotransmission in some brain sites. These pathways exist through direct binding to specific membrane receptors or through an indirect action via genetic mechanisms and differences in gene expression and protein content (Wilson and Westberry 2009). The regulation of pituitary gonadotropin secretion may result from a complex interaction between a gonadal steroid feedback mechanism and the influence of brain neurotransmitter systems in the hypothalamic–pituitary system, including the opioidergic system (Kalra 1993; Wójcik-Gładysz et al. 2006; Kaminski et al. 2004; Yilmaz and Gilmore 2000).

Endogenous opioids can modulate the catecholamine pathway, inhibit catecholamine release during times of stress, and inhibit gonadotropin secretion (Angelopoulos et al. 1995). Opioid receptors can have various effects that depend on their localization in the brain. The analgesic activity of these receptors has been observed in the spinal cord and brainstem. In regions of the striatum and hypothalamus, they appear to modulate motor and reproductive function, respectively (Hammer et al. 1994; Teodorov et al. 2006, 2014; Miranda-Paiva et al. 2007; Yim et al. 2006; Sukikara et al. 2006).

The opioidergic system plays roles at both central and peripheral sites. At central sites, the opioidergic system plays a critical role in reproductive neuroendocrine function, whereas peripheral actions on carbohydrate metabolism and insulin resistance have been reported (Fulghesu et al. 2001). β-endorphin plays a modulatory role by inhibiting gonadotropin-releasing hormone and luteinizing hormone (LH) release from the pituitary. Other studies showed that the increase in opioidergic system activity in patients with polycystic ovary syndrome was suppressed by opioidergic antagonist administration (Zangeneh et al. 2011). Indeed, κ- and δ-opioid receptors have been well studied and appear to be associated with the control of gonadotropin secretion (Pfeiffer et al. 1987; Micevych et al. 2003; Sinchak et al. 2004).

Few studies have investigated the role of opioid receptor subtypes in different regions of the rat brain with regard to how steroid hormones act in this context. Important studies have been conducted with regard to μ-opioid receptors. The number of these receptors in the rat brain was found to fluctuate during different phases of the estrous cycle (Casulari et al. 1987; Stoffel et al. 2005), and this phenomenon could explain the different pathways of the effects that modulate the release of gonadotropins by opioidergic agonists and antagonists during different phases of the estrous cycle (Kalra et al. 1993; Piva et al. 1985).

The present study evaluated the modulatory role of morphine and steroid hormones in ovariectomized (OVX) virgin rats with regard to *Oprm1*, *Oprk1*, and *Oprd1* gene expression in the PAG, striatum, and hypothalamus because these regions are involved in the control of motivated, motor, and reproductive behaviors.

## Results

**Figure 1:**
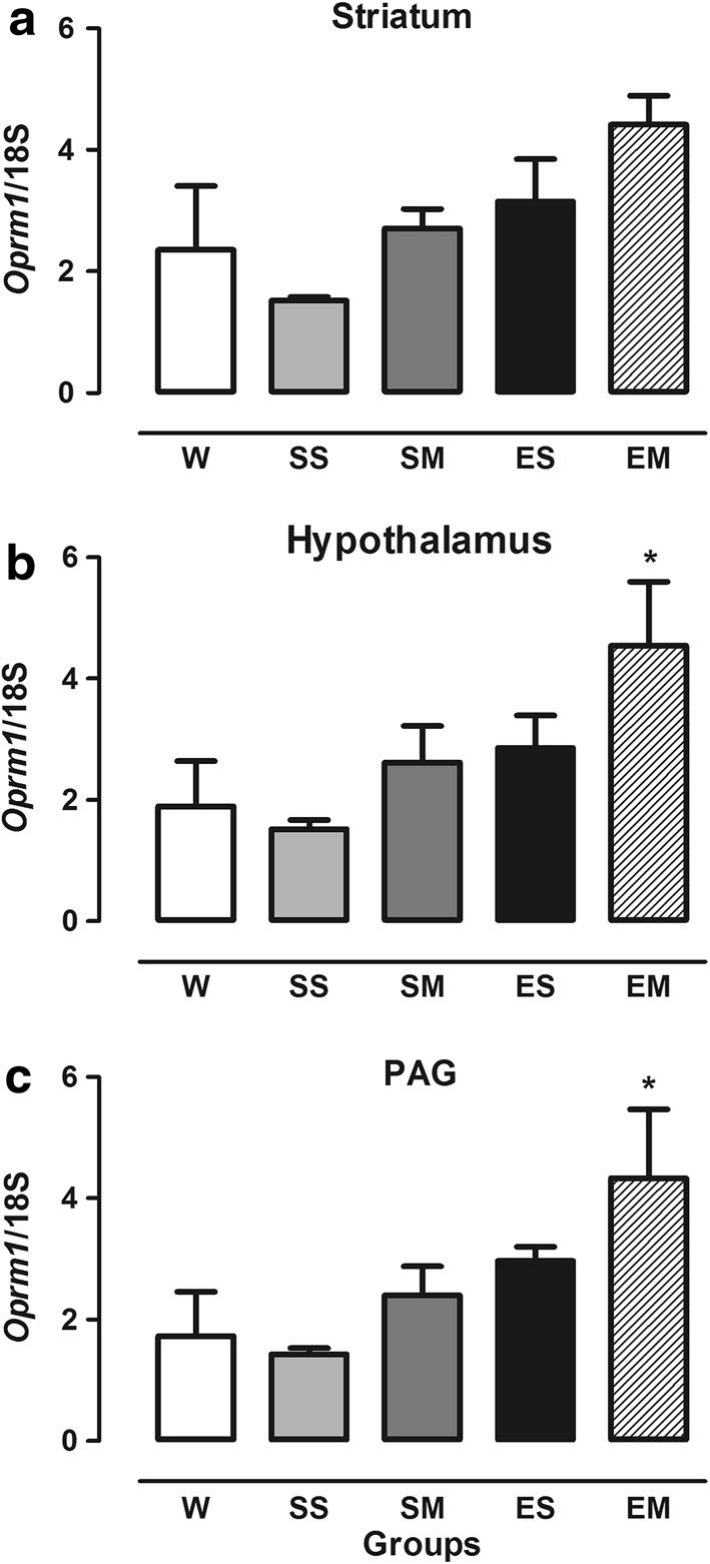
Expression of *Oprm1* gene in the striatum (**a**), hypothalamus (**b**), and PAG (**c**) in OVX female rats. The W group remained undisturbed. The SS group received saline solution 54 and 2 h before the experiments. The SM group received saline 54 h and 3.5 mg/kg morphine 2 h before the experiments. The ES group received estrogen (50 µg/kg, i.p.) 54 h before testing and saline 2 h before the experiments. The EM group received estrogen (50 µg/kg, i.p.) 54 h before testing and 3.5 mg/kg morphine 2 h before the experiments. *p < 0.05, compared with SS group. The data are expressed as mean ± SEM (ANOVA followed by Tukey’s multiple-comparison test).

*Oprm1* gene expression (Figure 1) was significantly increased in the EM group in the hypothalamus (*F* = 2.98, df = 15, *p* = 0.05; Figure 1b) and PAG (*F* = 3.112, df = 15, *p* = 0.047; Figure 1c) compared with the SS group. No significant differences in *Oprm1* gene expression were found in the striatum between groups (*F* = 0.379, df = 15, *p* = 1.122; Figure 1a).

**Figure 2:**
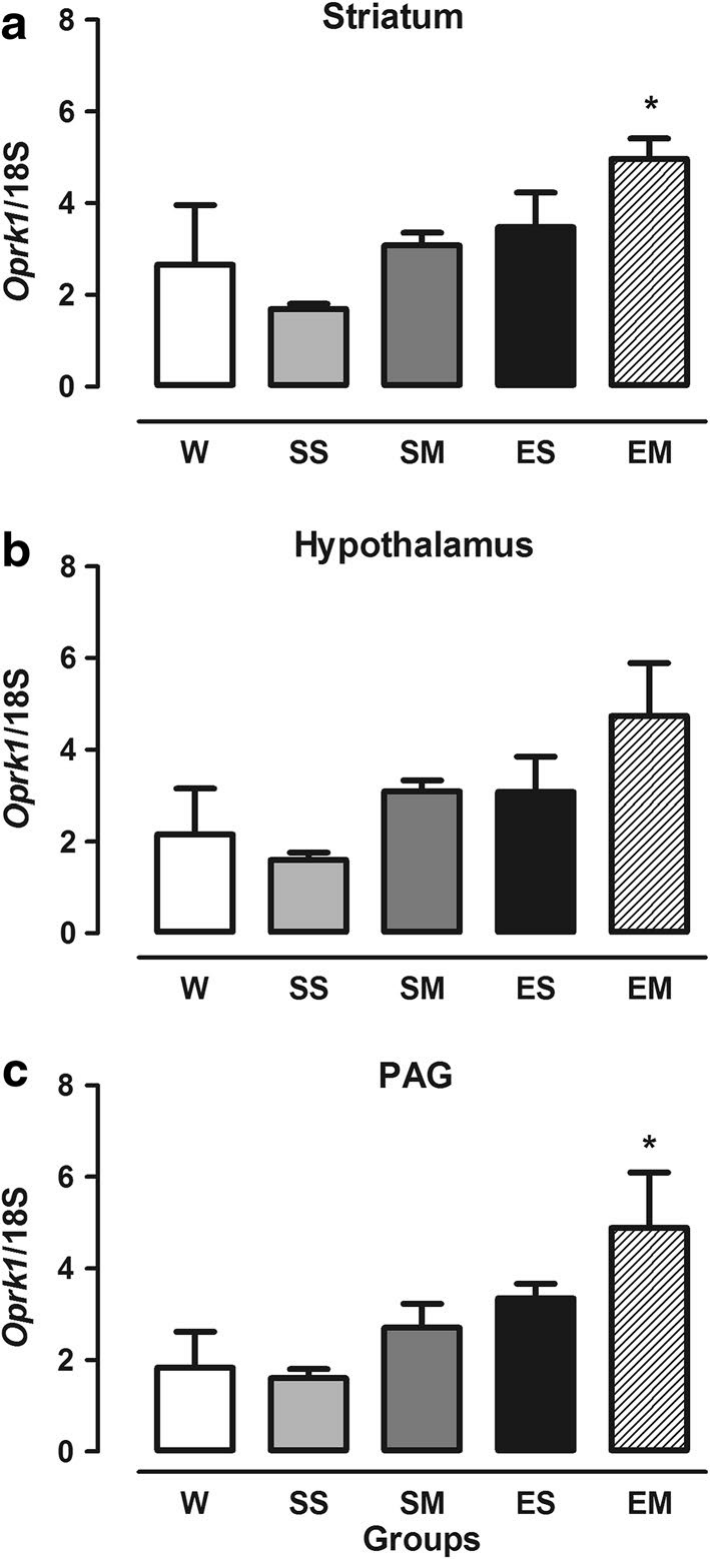
Expression of *Oprk1* gene in the striatum (**a**), hypothalamus (**b**), and PAG (**c**) in OVX female rats. The W group remained undisturbed. The SS group received saline solution 54 and 2 h before the experiments. The SM group received saline 54 h and 3.5 mg/kg morphine 2 h before the experiments. The ES group received estrogen (50 µg/kg, i.p.) 54 h before testing and saline 2 h before the experiments. The EM group received estrogen (50 µg/kg, i.p.) 54 h before testing and 3.5 mg/kg morphine 2 h before the experiments. *p < 0.05, compared with SS group. The data are expressed as mean ± SEM (ANOVA followed by Tukey’s multiple-comparison test).

*Oprk1* gene expression (Figure 2) was significantly increased in the EM group in the striatum (*F* = 2.855, df = 15, *p* = 0.061; Figure 2a) and PAG (*F* = 3.353, df = 15, *p* = 0.032; Figure 2c) compared with the SS group. No differences in *Oprk1* gene expression were found in the hypothalamus between groups (*F* = 2.383, df = 15, *p* = 0.078; Figure 2b).

**Figure 3:**
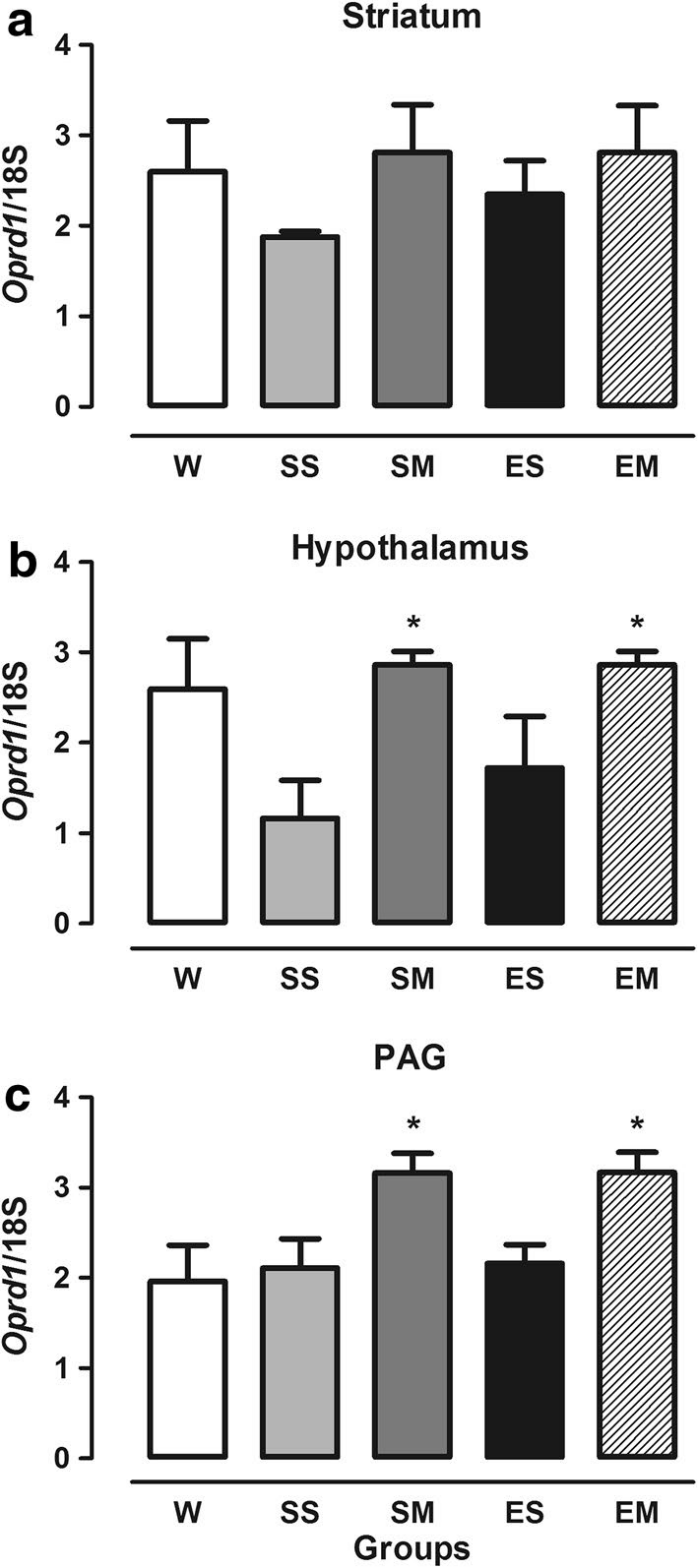
Expression of *Oprd1* gene in the striatum (**a**), hypothalamus (**b**), and PAG (**c**) in OVX female rats. The W group remained undisturbed. The SS group received saline solution 54 and 2 h before the experiments. The SM group received saline 54 h and 3.5 mg/kg morphine 2 h before the experiments. The ES group received estrogen (50 µg/kg, i.p.) 54 h before testing and saline 2 h before the experiments. The EM group received estrogen (50 µg/kg, i.p.) 54 h before testing and 3.5 mg/kg morphine 2 h before the experiments. *p < 0.05, compared with SS group. The data are expressed as mean ± SEM (ANOVA followed by Tukey’s multiple-comparison test).

*Oprd1* gene expression (Figure 3) was significantly increased in the SM and EM groups in the hypothalamus (*F* = 0.757, df = 15, *p* = 0.569; Figure 3b) and PAG (*F* = 4.473, df = 15, *p* = 0.014; Figure 3c) compared with the SS group (Figure 1c). No differences in *Oprd1* gene expression were found in the striatum between groups (*F* = 0.757, df = 15, *p* = 0.569; Figure 3a).

## Discussion

In rodents, hormone fluctuations characterize each estrous phase as proestrus, estrus, metestrus, and diestrus. Studies have shown that a low level of estrogen during diestrus has an inhibitory action on the hypothalamus and pituitary (Kirkpatrick and Clark 2011; Piva et al. 1985), and gonadotropin release does not occur (Danforth et al. 1990). A gradual increase in estrogen levels is observed during the late stage of diestrus. In proestrus, a rapid increase in estrogen and progesterone levels occurs, resulting in LH release that enables ovulation (Gómez-Camarillo et al. 2011; Gordon et al. 2011; Alvarenga et al. 2010).

The present study investigated the role of acute estrogen administration on opioid gene expression in the striatum, hypothalamus, and PAG and the effects of acute morphine treatment. To avoid the possible effects of endogenous ovarian hormones, we used OVX rats and subjected them to experimentation after 15 days to elicit total hormone depletion (Rachman et al. 1998). Importantly, adrenal hormones were not considered in this study. Thus, OVX female rats were used as an animal model to study the influence of estrogen replacement on the activity of the genes that encode opioid receptors in female rats in response to morphine in different brain areas. Studies have shown that the restoration of reproductive behavior and changes in gene expression in OVX rats occur within 7 days of continuous estrogen replacement, supporting the procedure used in the present study with regard to the hormonal depletion protocol followed by replacement. The doses of morphine and estrogen hormones used in this study were previously validated in our laboratory and standardized for behavioral and molecular analyses (Teodorov et al. 2006, 2011, 2014).

Studies have evaluated the density of μ-, κ-, and δ-opioid receptors in many brain structures in rats (Mansour et al. 1994, 1995), demonstrating a basal pattern. A high density of μ-opioid receptors is found in the PAG. A moderate density is found in the striatum, and less expression is found in the hypothalamus. A high density of κ-opioid receptors is found in the hypothalamus, with less expression in the striatum and PAG. A high density of δ-opioid receptors is found in the striatum, with less density in the PAG and hypothalamus.

Weiland and Wise (1990) showed that steroid hormones modulate opioid receptor density in specific brain regions. Their results showed negative and positive feedback actions of estrogen and progesterone in hypothalamic regions that regulate gonadotropin release and sexual behavior. These actions may be partially mediated by the endogenous opioid system. Estrogen and progesterone suppress physiological responses to the administration of opioid peptides.

The present study evaluated the basal pattern of *Oprm1*, *Oprk1*, and *Oprd1* gene expression in female rat brain sites that are known to be involved in reproductive behavior, including the striatum (i.e., important role in motor patterns during mating and lactation), PAG (i.e., role in nociception, behavioral selection for motivated behaviors, and sexual and maternal behavior), and hypothalamus (i.e., role in the modulation of hormonal aspects) (Sewards and Sewards 2003; Moura et al. 2010; Mota-Ortiz et al. 2012; Teodorov 2012). Studies have shown that estrogen is able to change the action of the opioidergic system in rodents by modulating nociceptive responses to treatment with naloxone, altering the binding of opioid receptors in specific brain regions, and increasing the rate of internalization of these receptors, particularly in morphine studies (Hammer 1990; Gordon and Soliman 1996; Aloisi and Ceccarelli 1999; Micevych et al. 2003). Terner et al. (2005) showed that morphine and the semi-synthetic opioid derivative buprenorphine dose-dependently increased the patterns of nociception in rats, and morphine exerted these effects during all phases of the estrous cycle, whereas buprenorphine was effective only during estrus.

In the present study, *Oprm1* gene expression in the hypothalamus and PAG was activated by morphine only in estrogen-treated animals. *Oprk1* gene expression in the striatum and PAG was activated by morphine only in estrogen-treated animals. *Oprd1* gene expression in the hypothalamus and PAG was activated by morphine in both estrogen-treated and -nontreated animals. These results suggest that the *Oprm1* and *Oprk1* gene responses to morphine might depend on estrogen, whereas the *Oprd1* gene response to morphine might not depend on estrogen. On this respect, κ receptors of the PAG have been show to be functionally involved in motivated behaviors during the post-partum phase (Mota-Ortiz et al. 2012; Teodorov 2012). Recently, a functional interaction between μ and κ opioid receptor subtypes has been demonstrated (Klein et al. 2014). Since, this finding was observed in a post-partum scenario, the fact that those receptors subtypes were found to be particularly sensitive to estrogen modulation actions might be another piece of these puzzle that tell us about the multiple functional interactions that those to versatile opioid receptor subtypes. These data support the hypothesis of a functional role for ovarian hormones in opioid receptor-mediated functional adaptations in the female brain.

## Methods

### Animals

Female Wistar rats, weighing 200–250 g at the beginning of the study, were obtained from the Faculdade de Medicina Veterinária, Universidade de São Paulo. The animals were housed in polypropylene cages (32 × 40 × 18 cm), with three animals per cage under controlled temperature (22 ± 2°C) and a 12 h/12 h light/dark cycle (lights on 6:00 AM) with free access to food and water during the experimental procedure. We attempted to minimize the number of rats used, and every effort was made to ensure that no rat suffered unnecessarily.

### Drugs

Morphine sulfate (3.5 mg/kg; Cristalia) was diluted in 0.9% saline, and the same vehicle was used as a control group. The hormones (progesterone and estradiol benzoate; Sigma-Aldrich (Brazil) were diluted in peanut oil. All of the injections were administered subcutaneously in the dorsal region of the animals.

### Molecular studies

RNA extraction, cDNA synthesis, and real-time polymerase chain reaction (PCR) were used for the quantification of opioid receptor gene expression. Total RNA was extracted from each tissue sample using Trizol reagent (Invitrogen Life Technologies, Carlsbad, CA, USA). Immediately after euthanasia by decapitation, the striatum was suspended in 1 ml ice-cold Trizol, and total RNA was extracted according to the manufacturer’s instructions. This area was chosen because it modulates motor function and opioid dependence (Thompson et al. 2000; Georges 1999) and has a high density of opioid receptors. The final RNA pellets were resuspended in 50 μl of diethyl pyrocarbonate-treated water. The total RNA concentrations were measured spectrophotometrically at 260 nm, and the integrity of the RNA samples was analyzed on 1.5% agarose gel (Sigma, St. Louis, MO, USA) that contained 0.5 μg/ml ethidium bromide. Total RNA was then treated with DNAse I and stored at −80°C until further processing. Real-time PCR was performed in a Rotor Gene 3000 instrument (Corbet) using TaqMan Universal Master Mix (Applied Biosystems, catalog no. 4304437). The PCR primers and TaqMan probes for the opioid receptor genes *Oprm1*, *Oprk1*, and *Oprd1* were selected using Primer Express software (Applied Biosystems), verified by a BLAST search of GenBank, and labeled Rn00565144_m1 for *Oprm1*, Rn00567737_m1 for *Oprk1*, RN00561699_m1 for *Oprd1*, and 4319413E for 18S (used as a housekeeping control). The primers were chosen to amplify a 65-bp fragment. The internal TaqMan probe [FAM-3′-TCTGGGCACCTCTCTTT-5′-non-fluorescent quencher (NFQ)] was designed according to the general rules outlined by the manufacturer and carried a 5′ reporter dye, 6-carboxy fluorescein (FAM), and 3′ NFQ. The primers and probes were used with 100% efficiency at final concentrations of 0.9 and 0.25 μM, respectively.

### Experimental design

A total of 20 female rats were observed by vaginal cytology for 15 days, always in the morning, to verify that all of the female rats were cycling normally. After the vaginal cytology tests, these rats were ovariectomized between 8:00 AM and 10:00 AM and remained undisturbed for 15 days to deplete hormone stores (Vignon and Rochefort 1976). These OVX female rats were then divided into four equal groups. Two groups received estrogen (50 µg/kg, i.p., 54 h before testing) and saline (ES group, n = 4) or 3.5 mg/kg morphine (i.p.; EM group, n = 4) 2 h before euthanasia. The other two groups received saline solution 54 and 2 h before saline (SS group, n = 4) or 3.5 mg/kg morphine (SM group, n = 4) treatment. *Oprm1, Oprd1*, and *Oprk1* gene expression was evaluated in the striatum, hypothalamus, and PAG. An OVX group, called white, remained untreated (W group, n = 4).

### Statistical analysis

The results are expressed as mean ± SEM. Homoscedasticity was verified using an F test or Bartlett’s test. Normality was verified using the Kolmogorov–Smirnov test. Differences in scores between more than two groups were assessed by one-way analysis of variance (ANOVA) followed by Tukey’s multiple-comparison test. The results were considered significant at *p* < 0.05.

## Conclusions

In this study we evaluated the effects of acute treatment with morphine on the expression of the *Oprm1*, *Oprk1*, and *Oprd1* opioid genes in some cerebral areas that could be modulated by sexual hormones in ovariectomized female rats. The results showed that these genes expression could be changed in response to morphine, especially by estrogen dependence, supporting the hypothesis of a functional role of ovarian hormones in opioid receptor-mediated functional adaptations in the female brain. This is very important because whereas this study used ovariectomized female rats, the use of opioid analgesics in intact females could change parameters of reproduction and promote a injury in maternal homeostasis, that could influence parental care and generate alterations in offspring in adulthood life.
